# A comprehensive study of immunology repertoires in both preoperative stage and postoperative stage in patients with colorectal cancer

**DOI:** 10.1002/mgg3.504

**Published:** 2019-01-09

**Authors:** Xicheng Liu, Yuanyuan Cui, Yaoxian Zhang, Zhanli Liu, Qiuli Zhang, Wenyan Wu, Zihao Zheng, Shien Li, Zhongjun Zhang, Yali Li

**Affiliations:** ^1^ Department of Anesthesiology, Shenzhen People’s Hospital 2nd Clinical Medical College of Jinan University Shenzhen Guangdong China

**Keywords:** CDR3, CEGA, CRC, repertoire, TCRs, TIVA

## Abstract

**Background:**

Colorectal cancer (CRC) is the 3rd most common cancer type in the world. The correlation between immune repertoire and prognosis of CRC has been well studied in the last decades. The diversity and stability of the immune cells can be measured by hypervariable complementarity‐determining region 3 (CDR3) segments of the T‐cell receptor (TCR).

**Methods:**

In this study, we collected five healthy controls and 19 CRC patients’ peripheral blood mononuclear cells (PBMCs) in three stages, namely 1 day preoperative, 3 days’ postoperative, and 7 days’ postoperative, respectively. Simultaneously, we have also done the comparative analysis of these two different anesthesia methods, namely TIVA and CEGA. Sequencing of the TCR segments has been performed by multiplex PCR and high‐throughput next‐generation sequencing. We also analyzed the distribution of CDR3 length, highly expansion clones (HECs), TRBV, and TRBJ gene usage.

**Results:**

Our result showed a significant difference between TCR CDR3 length distribution and HEC distribution between CRC patients and healthy controls. We also found that TRBV11‐2, TRBV12‐1, TRBV16, TRBV3‐2, TRBV4‐2, TRBV4‐3, TRBV5‐4, TRBV6‐8, TRBV7‐8, TRBV7‐9 and RBV11‐2, TRBV12‐1, TRBV16, TRBV3‐2, TRBV4‐2, TRBV4‐3, TRBV5‐4, TRBV6‐8, TRBV7‐8, and TRBV7‐9 usages are different between CRC patients and healthy controls.

**Conclusion:**

In conclusion, CRC patients were presented with different immune repertoire in comparison with healthy controls. In this study, significant difference in TRBV and TRBJ gene usage in between case and control group could provide some potential biomarker for the diagnosis and the treatment of the patients with CRC.

## 1 INTRODUCTION

Colorectal cancer (CRC) is the third most common cancer in the world and the fifth most prevalent cancer type in China, causing 376.3 thousand new patients and 191.0 mortality a year (Chen, Xu, et al., 2016a; Chen, Zheng, et al., 2016b; Wang et al., 2017). The occurrence of CRC is directly correlated with the abnormal immunological microenvironment. It has been reported that the immunotherapy on cancer, including CRC, is very effective, which allows us to investigate the immune repertoire study in CRC patients (Hope et al., 2017). Further study on CRC patients in respect to change in immunological microenvironment with origin as well as the prognosis of cancer is a one of the significant methods for early detection of biomarkers as well as identifying the target for immunotherapy. It is well studied that the first‐tier treatment of colorectal cancer is timely surgical interventions or total colectomy (Adelson et al., 2018). Hence, different anesthesia pattern would have a significant role for the prognosis of colorectal cancer. Till date, there is no study has been performed for the comparison of colorectal cancer patients and healthy controls’ in terms of TRCs and the different methods of anesthesia.

T cells are the active cell population‐mediating cellular immune response and function as an important component in humoral immune system activation response. T‐cell receptors (TCRs) are the antigen recognition part on T‐cell membrane, which is composed of α and β chain, or γ and δ chain. Complementarity‐determining region 3 (CDR3) is a critical region in TCR on both the chains, responsible for specifically recognize and bind antigen peptide. Each T cell has its own unique CDR3 sequence. According to the homology of CDR3 variable region (V) gene sequence, TCR Vβ genes are divided into 24 families. Testing of each Vβ gene family's CDR3 spectra could reflect the clonal expansion of T cells (Luo et al., 2014).

In this study, we applied high‐throughput next‐generation sequencing (NGS) to elucidate the immune repertoire status among sporadic colorectal cancer patients (T1M0N0; Stage I) in different time points (1 day before surgery, 3 days’ after surgery, and 7 days after surgery) with different anesthesia methods to patients and healthy controls. Then, the distribution of CDR3 length in preoperative patients and healthy controls was studied. Additionally, highly expanded clone distribution in preoperative patients, healthy controls, and postoperative patients at different time points with different anesthesia (TIVA and CGEA) methods has been compared in this study. In order to understand the mechanism of CRC immune exchange, TRBV, TRBJ gene repertoires between preoperative patients and healthy controls would also be studied.

## 2 MATERIALS AND METHODS

### 2.1 Patients and controls

Whole blood samples from 19 CRC patients and five healthy controls were collected at The Second Medical College of Jinan University (Shenzhen People's Hospital), Shenzhen, China, and PBMCs were extracted. We collected the PMBCs of 10 colon cancer patients, who had taken the TIVA anesthesia pattern, at 1 day preoperative, 3 days’ postoperative, and 7 days’ postoperative time point, respectively. The PMBCs of nine CRC patients, who had taken the CEGA anesthesia, at 1 day preoperative, 3 days’ postoperative, and 7 days’ postoperative time point were collected. The Ethical Committee of the Department of Anesthesiology, Shenzhen People's Hospital, 2nd Clinical Medical College of Jinan University, Shenzhen, Guangdong, China, reviewed and approved our study protocol in compliance with the Helsinki Declaration.

### 2.2 T‐cell isolation and DNA extraction

Informed consent was obtained from all the participants in our study. T‐cell isolations were performed using superparamagnetic polystyrene beads (Miltenyi) coated with monoclonal antibodies specific for T cells. DNA was prepared from 0.5 to 2 × 106 T cells from each sample, which was sufficient for analyzing the diversity of TCRV in the T‐cell subsets. DNA was extracted from PBMCs using GenFIND DNA (Agencourt, Beckman Coulter, Brea, CA, USA) extraction kits following the manufacturer's instructions.

### 2.3 Multiplex‐PCR amplification of the TCR CDR3 region

The TCR CDR3 region was defined according to International ImMunoGeneTics collaboration, starting with the second conserved cysteine encoded by the 39 portions of the V gene segment and ending with the conserved phenylalanine encoded by the 59 portions of the J gene segment. To generate the template library for Genome Analyzer, a multiplex‐PCR system was designed to amplify rearranged TCR CDR3 regions from genomic DNA using 45 forward primers, each is specific to a functional TCR V segment, and 13 reverse primers, each is specific to a TCR J segment. The forward and reverse primers contain, at their five ends, the universal forward and reverse primer sequences, respectively, which are compatible with GA2 cluster station solid‐phase PCR. After amplification and selection, the products were purified using QIAquick PCR Purification Kit. The final library was quantitated in two ways: by determining the average molecule length using the Agilent 2100 bioanalyzer instrument (Agilent DNA 1000 Reagents) and by real‐time quantitative PCR (QPCR; TaqMan Probe). The libraries were amplified with cBot to generate the cluster on the flow cell, and the amplified flow cell was pair‐end (PE) sequenced using a Hiseq2500 instrument, with a read length of 100 as the most frequently used sequencing strategy.

### 2.4 High‐throughput sequencing and data analysis

The PCR products were sequenced using an Illumina Genome Analyzer, and the sequencing quality of these reads was evaluated by the formula shown below. The quality of the HiSeq sequencing ranged from 0 to 40 and was used for filtering out low‐quality reads. First, we filtered the raw data, including adapter contamination. Reads with an average quality score lower than 15 (Illumina 0–41 quality system) were removed, and the proportion of N bases was not more than 5% (sequences with higher values were also removed). Next, a few bases with low quality (lower than 10) were trimmed; the quality score was expected to be over 15 after trimming, and the remaining sequence length was expected to be more than 60 nt. After filtering, PE read pairs were merged into one contig sequence in two steps: (a) by aligning the tail parts of two sequences and assessing the identity (BGI developed software COPE v1.1.3), with at least 10 bases of overlap required and the overlapping section having 90% base match; (b) as different primers might result in sequences of different lengths, some might be very short (<100 bp) and such reads were merged by aligning the head part of the sequence (BGI developed software FqMerger). In this way, we obtained the merged contig sequences and the length distribution plot. Subsequently, we used miTCR, developed by MiLaboratory (https://mitcr.milaboratory.com/downloads/) for the alignment. This program has an automated adjustment mechanism for errors introduced by sequencing and PCR and will provide alignment statistical information, such as the CDR3 expression and INDEL. After alignment, we utilized the following method for the sequence structural analysis: (a) We calculated the number of each nucleotide and analyzed the proportion at each position; (b) according to the last position of the V gene, start site of the D gene, end site of the D gene, and start site of the J gene after alignment, we retrieve the INDEL (insertion and deletion) introduced during V–D–J recombination; (c) nucleotides were translated into amino acids. According to the identity of each sequence after alignment, the expression level of each clone was clear and calculated. The expression of each distinct DNA sequence, amino acid sequence, and V–J combination was also identified. In addition, to measure the diversity of each sample, we calculated the distinct clone number, Simpson coefficient, and Shannon–Waver coefficient based on different resolutions of distinct DNA sequences, amino acid sequences, and V–J combinations. The expression level of each sample was also calculated at different resolutions of distinct DNA sequence, amino acid sequence, and V–J combination. Moreover, we constructed the specific expression graph and plotted a heatmap according to the V–J combination profile. The diversity of the TCR repertoire was calculated based on the Simpson index of diversity (Ds) and the Shannon–Wiener index (*H*).

### 2.5 Statistical analysis

Because of the small sample size in this study, the analysis of differences among the data groups was performed with the *t* test. *p* values <0.05 were considered significant. The statistical analyses were conducted with GraphPad Prism software (GraphPad Software, San Diego, CA, USA).

## 3 RESULTS

### 3.1 Sequencing data summary

**Figure 1:**
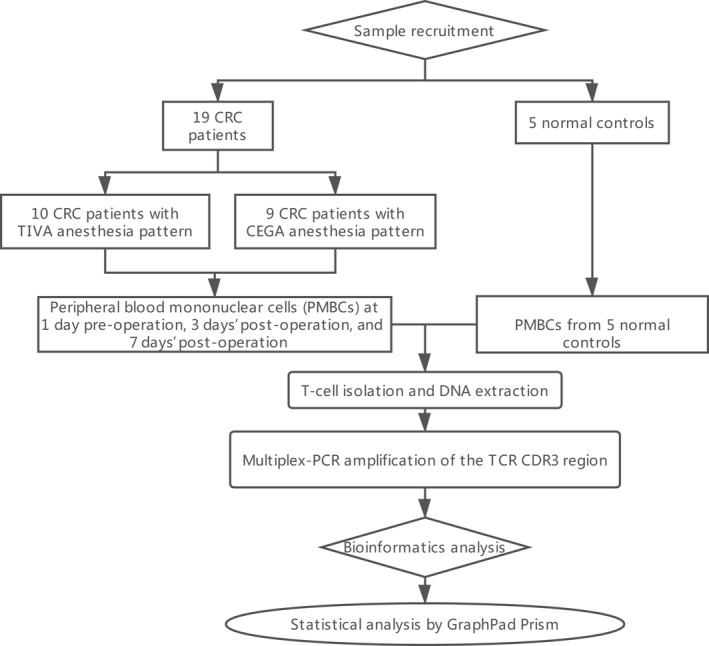
The details of data interpretation pipeline

A total of 19 colorectal cancer patients and five healthy controls were recruited for this study. Blood sample from 1 day preoperative, 1 day postoperative, 3 days’ postoperative, and 7 days’ postoperative was collected. We obtained an average of 713,362 sequencing reads per sample (Figure 1). The mean unknown sequence number is 15,856, and the mean immune sequence number is 697,505. The productive sequence number and the nonproductive sequence number are 519,165 and 178,340, respectively. And *In‐frame* sequences number and *Out‐of‐frame* sequences number are 539,825 and 151,114, respectively. The total CDR3 sequence number, unique CDR3 nt sequence number, and Unique CDR3 aa sequence number are 505,707, 54,921, and 47,892 respectively (Table 1).

**Table 1:** The sequencing data of preoperation, postoperation colorectal cancer (CRC) patients, and normal controls

Sample	Total read number	Immune sequence number	Unknown sequence number	Productive sequence number	Nonproductive sequence number	In‐frame sequence number	Out‐of‐frame sequence number	Total CDR3 sequence number	Unique CDR3 nt sequence number	Unique CDR3 aa sequence number
AC1	1,024,851	1,018,261	6,590	763,607	254,654	791,978	222,451	749,378	51,515	44,194
AC2	915,539	907,787	7,752	612,514	295,273	636,437	267,323	594,790	23,903	19,362
AC3	816,031	809,723	6,308	596,926	212,797	623,456	182,764	585,065	34,505	28,231
AD1	585,142	579,428	5,714	434,691	144,737	451,489	124,876	423,863	42,681	37,572
AD2	472,688	467,463	5,225	308,195	159,268	322,541	142,372	296,522	21,470	18,205
AD3	491,946	487,345	4,601	358,566	128,779	372,844	111,952	347,880	35,859	31,372
AE1	689,228	686,592	2,636	515,251	171,341	536,573	148,702	506,671	44,802	38,574
AE2	652,635	648,916	3,719	454,498	194,418	473,915	173,199	445,088	25,864	21,307
AE3	618,329	615,743	2,586	467,389	148,354	486,543	127,915	459,618	40,967	35,152
AF1	638,916	635,461	3,455	480,936	154,525	507,267	126,411	459,509	57,346	50,990
AF2	576,782	574,195	2,587	413,875	160,320	435,968	136,955	393,742	27,561	22,786
AF3	556,098	553,235	2,863	411,855	141,380	432,611	119,126	393,503	37,868	32,556
AH1	684,804	679,686	5,118	508,178	171,508	525,236	151,717	500,960	32,634	28,647
AH2	612,272	607,843	4,429	418,215	189,628	431,472	174,073	411,668	13,414	11,262
AH3	553,362	548,243	5,119	401,398	146,845	416,356	129,142	394,970	26,871	22,970
AJ1	888,349	881,175	7,174	666,063	215,112	691,334	185,866	655,889	48,562	41,840
AJ2	571,779	565,678	6,101	415,792	149,886	430,800	131,830	407,950	23,184	19,397
AJ3	593,718	587,471	6,247	397,681	189,790	416,218	168,132	388,181	24,027	19,801
BC1	457,877	456,003	1874	365,329	90,674	381,349	73,765	359,382	29,517	25,085
BC2	493,920	491,375	2,545	378,912	112,463	395,000	95,022	370,358	22,213	18,557
BC3	635,015	631,465	3,550	486,999	144,466	508,131	121,623	478,226	28,547	23,598
BD1	668,144	664,884	3,260	540,400	124,484	558,052	105,180	531,880	18,627	15,004
BD2	517,884	514,866	3,018	374,193	140,673	391,179	122,250	366,294	18,125	14,729
BD3	444,360	440,878	3,482	292,794	148,084	304,829	134,361	284,279	14,105	11,501
BF1	495,271	490,136	5,135	381,063	109,073	398,525	89,012	373,918	35,855	31,343
BF2	553,048	546,105	6,943	347,777	198,328	367,448	175,352	335,777	22,811	18,999
BF3	929,050	922,164	6,886	687,932	234,232	722,551	196,043	674,703	37,951	31,204
BG1	653,871	650,887	2,984	499,435	151,452	519,916	129,541	491,033	39,035	33,055
BG2	555,950	549,055	6,895	328,977	220,078	344,390	201,461	317,677	21,045	17,857
BG3	455,728	449,852	5,876	257,201	192,651	271,112	175,899	246,750	17,082	14,160
BH1	969,580	963,580	6,000	758,935	204,645	787,303	173,146	746,555	43,678	36,545
BH2	808,748	803,701	5,047	638,897	164,804	661,579	139,513	629,097	35,323	29,200
BH3	586,924	580,323	6,601	398,425	181,898	413,850	163,541	387,228	18,139	14,569
BI1	577,915	574,247	3,668	458,549	115,698	474,530	97,725	441,438	38,294	32,526
BI2	471,440	466,428	5,012	343,870	122,558	356,712	107,489	328,694	20,062	16,528
BI3	583,748	576,360	7,388	400,607	175,753	417,237	155,274	379,554	36,364	31,090
HHT1	1,084,617	1,035,772	48,845	938,621	97,151	961,208	52,913	917,434	357,213	323,645
HHT2	1,040,725	987,555	53,170	891,697	95,858	913,539	50,317	869,464	377,925	343,288
HHT3	929,379	885,492	43,887	806,168	79,324	823,340	42,651	787,594	309,389	278,567
HHT4	2,146,006	1,877,696	268,310	1,186,570	691,126	1,242,699	547,083	1,129,853	54,996	44,183
HHT5	1,246,186	1,184,666	61,520	896,805	287,861	935,340	221,721	871,592	42,462	34,137
Average value	713362.3	697505.7	15856.59	519165.5	178340.2	539825.8	151114.3	505,708	54921.73	47892.39

Notes

AC1, AD1, AE1, AF1, and AJ1 represent sequencing data from PMBCs of CRC patients at 1 day preoperation in TIVA group. AC2, AD2, AE2, AF2, and AJ2 means sequencing data from PMBCs of CRC patients at 3 days’ postoperation in TIVA group. AC3, AD3, AE3, AF3, and AJ3 represent sequencing data from PMBCs of CRC patients at 7 days’ postoperation in TIVA group.

BC1, BD1, BF1, BJ1, BH1, and BI1 represents sequencing data from PMBCs of CRC patients at 1 day preoperation in CEGA group. BC2, BD2, BF2, BJ2, BH2, and BI2 represent sequencing data from PMBCs of CRC patients at 3 days’ postoperation in CEGA group. BC3, BD3, BF3, BJ3, BH3, and BI3 represent sequencing data from PMBCs of CRC patients at 7 days’ postoperation in CEGA group.

HHT1, HHT2, HHT3, HHT4, and HHT5 represent data from PMBCs of normal controls.

CDR3: complementarity‐determining region 3.

### 3.2 CDR3 length distribution pattern

**Figure 2:**
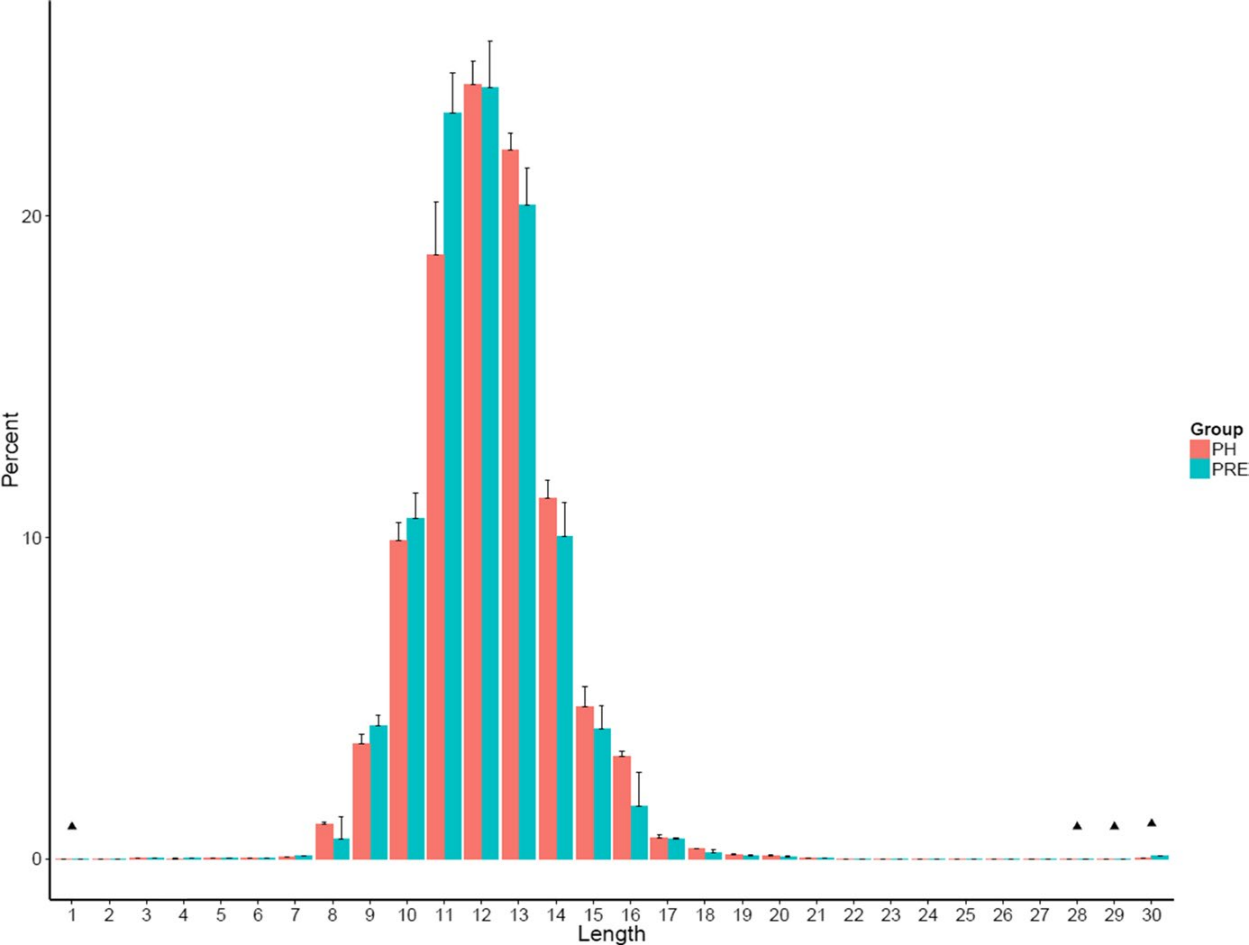
Complementarity‐determining region 3 length distribution in healthy controls and preoperation colorectal cancer (CRC) patients. “Pink” bar represents the value of healthy controls’, and “green” bar represents the value of preoperative CRC patients’. Black triangle represents the significant different (*p* < 0.05)

The length distribution of the TCR CDR3 is an important determinant of T‐cell repertoire diversity. In this study, we first assessed the length distribution of TCR CDR3 sequences (aa) in the preoperative group and healthy control group (Figure 2). TCR CDR3 sequence length in preoperative group was significantly higher compared to those in the NC group with the amino acid length 1 (*p* = 0.028), 28 (*p* = 0.026), 29 (*p* = 0.0064), and 30 (*p* = 0.00078).

**Figure 3:**
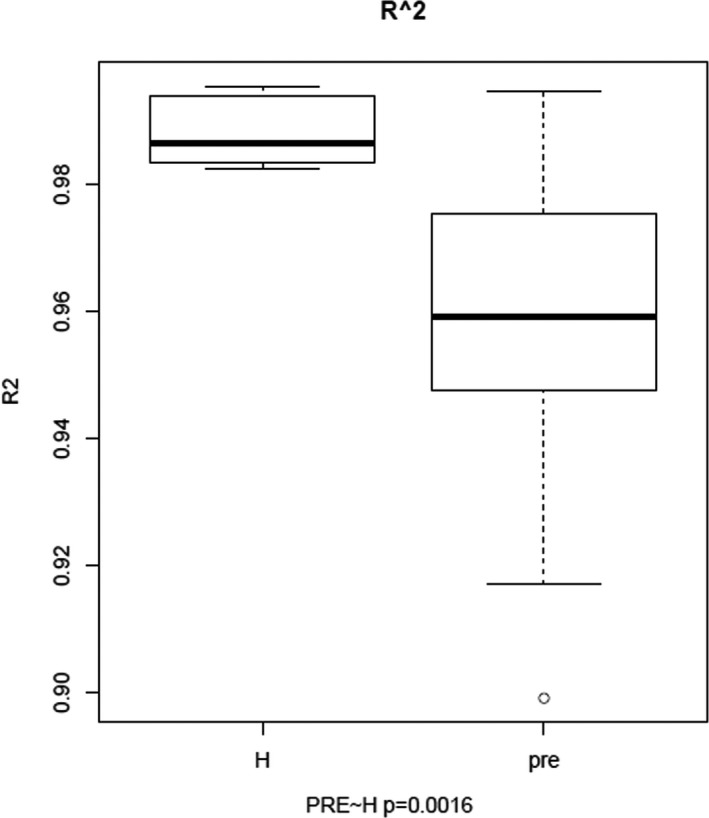
*R* ^2^ comparison between the preoperation colorectal cancer (CRC) patients and healthy controls. H represents value of the healthy controls, and pre represents value of the preoperation CRC patients

We draw the Gaussian distribution curve for each sample, and the goodness of fit was quantified by *R* ^2^, which ranges from 0 to 1 (from lowest fitness to highest fitness). *R* ^2^ values were calculated for each sample and compared between the preoperative CRC patients and healthy controls (*p* = 0.0016; Figure 3).

### 3.3 Highly expanded clones and TCR repertoires diversity

**Figure 4:**
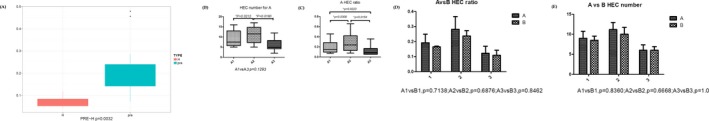
(a) Comparison of highly expansion clone (HEC) number distribution between healthy controls’ and preoperative colorectal cancer (CRC) patients’. H represents HEC number of healthy controls, and pre represents HEC number of preoperation CRC patients. (b) Comparison of HEC number of CRC patients who takes TIVA. A1 represents the value of PMBCs collected at 1 day preoperation. A2 represents the value of PMBCs collected at 3 days’ postoperation. A3 represents the value of PMBCs collected at 7 days’ postoperation. (c) Comparison of HEC ratio of CRC patients who takes TIVA. A1 represents the value of PMBCs collected at 1 day preoperation. A2 represents the value of PMBCs collected at 3 days’ postoperation. A3 represents the value of PMBCs collected at 7 days’ postoperation. (d) Comparison of HEC ratio between TIVA and CEGA groups at different time points. A represents TIVA groups, and B represents CEGA. Time line 1 represents the value of PMBCs taken from 1 day preoperation, 3 days’ postoperation, and 7 days’ postoperation. (e) Comparison of HEC ratio between TIVA and CEGA groups at different time points. (A) represents TIVA groups, (B) represents CEGA. Time line 1 represents the value of PMBCs taken from 1 day preoperation, 3 days’ postoperation, and 7 days’ postoperation

The expression level of each unique clone is another major measurement or index for immune diversity. After aligning to the human genome reference, the expression level of each clone is calculated. In this study, the TCR clones with frequency above 0.5% of total reads in a sample were defined as highly expansion clone (HEC). The comparison of HEC number between preoperative group and healthy control group showed significant higher HEC ratio in preoperative group (Figure 4a). In the TIVA group, HEC number of 3 days’ postoperative samples was higher than 1 day preoperative group (*p* = 0.021) and also higher than the value of 7 days’ postoperative group (*p* = 0.018; Figure 4b).

**Figure 5:**
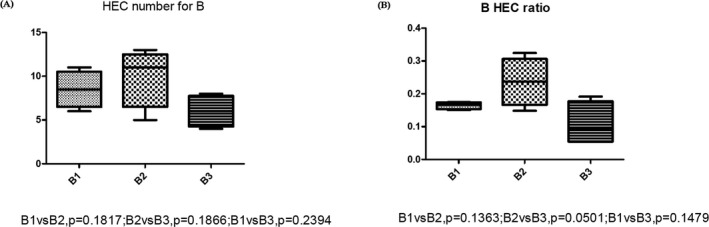
(a) Comparison of highly expansion clone (HEC) number of colorectal cancer (CRC) patients who takes CEGA anesthesia. A1, represents the value of PMBCs collected at 1 day preoperation. A2 represents the value of PMBCs collected at 3 days’ postoperation. A3 represents the value of PMBCs collected at 7 days’ postoperation. (b) Comparison of HEC ratio of CRC patients who takes CEGA anesthesia. A1 represents the value of PMBCs collected at 1 day preoperation. A2 represents the value of PMBCs collected at 3 days’ postoperation. A3 represents the value of PMBCs collected at 7 days’ postoperation

The HEC ratio of TIVA group also showed similar distribution of HEC number; the 3 days’ postoperative group showed higher HEC than 1 day preoperative group (*p* = 0.031), higher than the HEC ratio of 7 days’ postoperative group (*p* = 0.015). The HEC ratio of 7 days’ postoperative group was lower than 1 day preoperative group (*p* = 0.022; Figure 4c). To further study the difference in effect of TIVA and CGEA on immune repertoire, we also compared the HEC number and HEC ratio at the 3 different time period. The results showed there was no significant difference in the effect between the two anesthesia methods. (Figure 4d,e). The comparison of HEC number and HEC ratio at different time period in CGEA group showed no significant difference (Figure 5).

**Figure 6:**
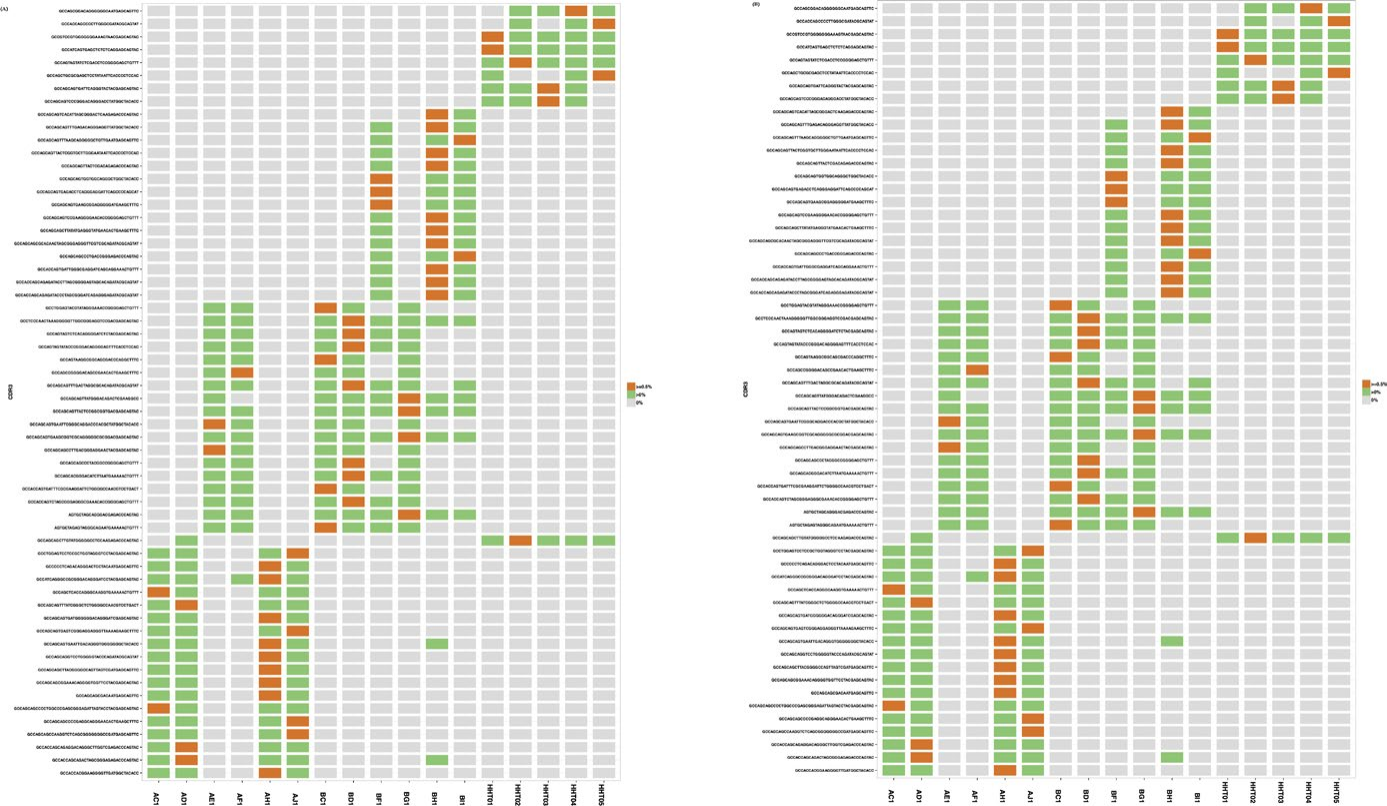
(a) Percentage of top 60 used complementarity‐determining region 3 (CDR3) nucleotides. AC1, AD1, AE1, AF1, AH1, AJ1, BC1, BD1, BF1, BG1, BG1, BH1, and BI1 are all preoperation colorectal cancer (CRC) patients. HHT01, HHT02, HHT03, HHT04, and HHt05 are all healthy controls. (b) Percentage of top 60 used CDR3 amino acids. AC1, AD1, AE1, AF1, AH1, AJ1, BC1, BD1, BF1, BG1, BG1, BH1, and BI1 are all preoperation CRC patients. HHT01, HHT02, HHT03, HHT04, and HHt05 are all healthy controls

To further understand the percentage of shared HECs, we then analyzed the top 60 highly used amino acids and nucleotide sequences in 0.5% used clones of CRC patients and healthy controls. According to Figure 6, there were highly shared sequences in CRC patients than in healthy controls.

### 3.4 Comparison of TRBV and TRBJ gene repertoires between preoperative patients and healthy controls

**Figure 7:**
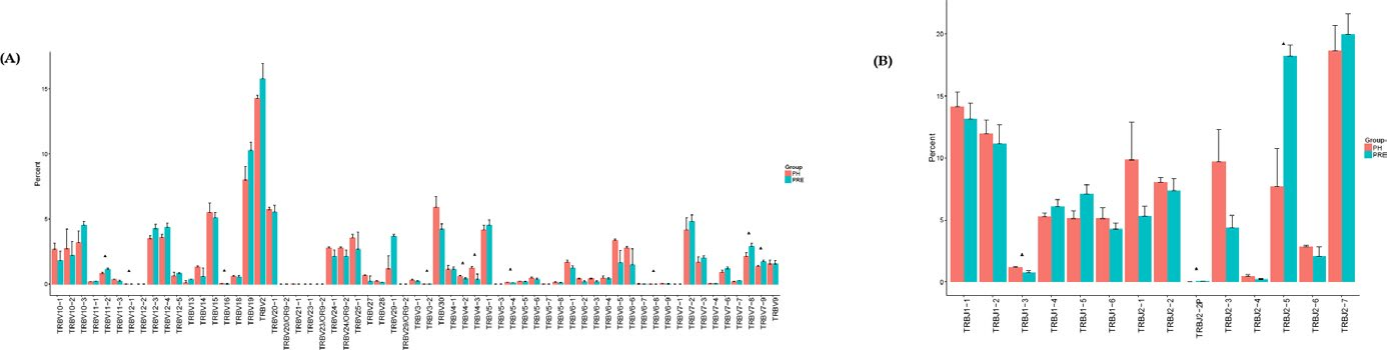
(a) Comparison of TRBV gene usage between preoperation patients and healthy controls. PH represents TRBV gene usage percentage of healthy controls, PRE, represents TRBV gene usage percentage of preoperation colorectal cancer (CRC) patients. Black triangle represents significant difference between healthy controls and preoperation CRC patients’ TRBV gene usage. (b) Comparison of TRBJ gene usage between preoperation patients and healthy controls. PH represents TRBV gene usage percentage of healthy controls, and PRE represents TRBV gene usage percentage of preoperation CRC patients. Black triangle represents significant difference between healthy controls and preoperation CRC patients’ TRBV gene usage

**Figure 8:**
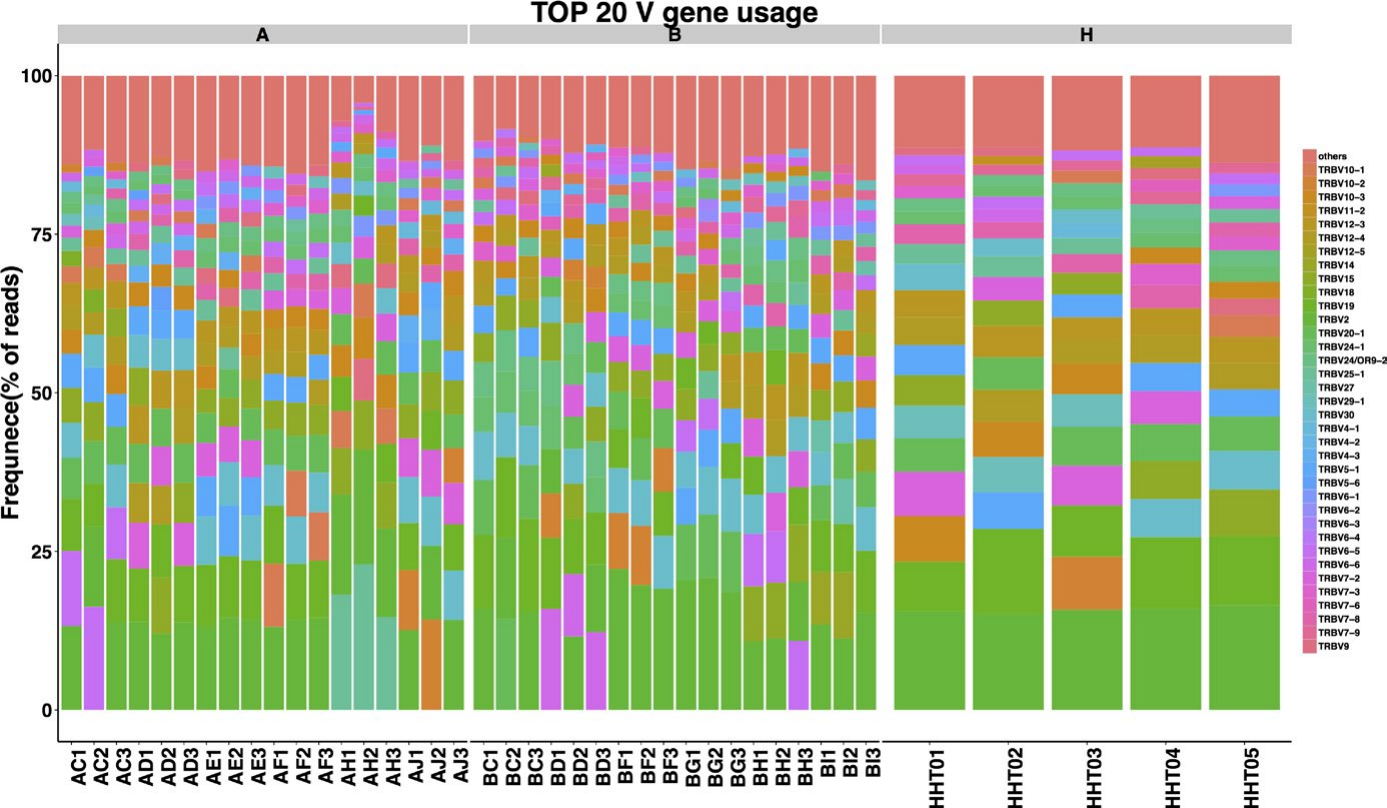
Heatmap of TOP 20 TRBV usage gene. A1 represents the value of PMBCs collected at 1 day preoperation. A2 represents the value of PMBCs collected at 3 days’ postoperation. A3 represents the value of PMBCs collected at 7 days’ postoperation

To determine the disease‐specific TCR repertoire characteristics, we compared the expression levels of TRBV and TRBJ genes of preoperative patients and healthy controls. In comparison with TRBV gene between preoperative patients and healthy controls, TRBV11‐2 (*p* = 0.016), TRBV12‐1 (*p* = 0.0068), TRBV16 (*p* = 0.0032), TRBV3‐2 (*p* = 0.0096), TRBV4‐2 (*p* = 0.03), TRBV4‐3 (*p* = 0.048), TRBV5‐4 (*p* = 0.011), TRBV6‐8 (*p* = 0.038), TRBV7‐8 (*p* = 0.042), and TRBV7‐9 (*p* = 0.023) usage showed significant difference (Figure 7a). In contrast, the differentiation of TRBJ gene between preoperative and healthy control patients showed significant difference in the usage of TRBJ1‐3 (*p* = 0.035), TRBJ2‐2 (*p* = 0.00053), and TRBJ2‐5 (*p* = 0.023; Figure 7b). Analysis of top 20 used TRBV genes was performed; TRBV7‐8, TRBV7‐9, and TRBV9 were well used in both CRC patients and healthy controls. TRBV2, TRBV12, TRBV19, TRBV20‐1, and TRBV24‐1 were poorly used in either CRC patients or healthy controls (Figure 8).

## 4 DISCUSSION

As the 3rd leading cause of tumor mortality, colorectal cancer is a well‐known and well‐studied type of cancer. The previous studies on colorectal cancer's biomarkers, surgery methods, metastatic mechanisms, target medicine‐related researches, anesthesia methods, immune repertoires, all have revealed the fundamental data (Daher, Chouillard, & Panis, 2014; Deschoolmeester, Baay, Specenier, Lardon, & Vermorken, 2010; Pan et al., 2017; Tsai et al., 2010). Here, we recruited 19 CRC patients and five healthy controls to study the difference in immune repertoire status among colorectal cancer patient at preoperative, postoperative, and healthy controls.

In comparison with CDR3 length distribution between preoperative colorectal patients and healthy controls showed there was significant difference between these two groups. This result again elucidated the immune repertoire effect on colorectal cancer patients which corresponds to the similar finding of previously reported CRC immune correlation study 1 (Li et al., 2016; Nakanishi et al., 2016).

Another well used factor to evaluate the immune repertoire status is HECs; the comparison between preoperative group and healthy control group again showed the significant higher HEC ratio and HEC number in CRC patients than the healthy control group, which could be the result of cancer‐immune reaction (Chen, Xu, et al., 2016a; Chen, Zheng, et al., 2016b). In addition, we found that TIVA patient group has significantly different HEC numbers and HEC ratios at different time period (1 day preoperative, 3 days’ postoperative, and 7 days’ postoperative), which could be a milestone for understanding and the management of the postoperation medical care. However, there were no differences in CGEA patient groups at different time period. Although there is possible effect of surgery on patients’ immune system, the difference in distribution of TIVA and CEGA on 3 days’ postsurgery and 7 days’ postsurgery patient group could provide more solid evidence to prove CEGA's potential advantage on immune repertoire balance. Then, the further hypothesis is to prove CGEA anesthesia has less effect on patients’ immune repertoires than the TIVA anesthesia or not despite the limited research samples, this could be a prestudy to further elucidate the effect of TIVA and CGEA on immune repertoires.

The random assortment of the V, (D), J gene segments provides the basic structural frames for antibody variable region to recognize specific antigen. Till now, only few experiments have performed the usage feature of V, (D), J gene segments. In the present study, all TRBV and TRBJ genes were deeply sequenced to study the potential specific higher usage. Between the patients and healthy control groups, TRBV11‐2 (*p* = 0.016), TRBV12‐1 (*p* = 0.0068), TRBV16 (*p* = 0.0032), TRBV3‐2 (*p* = 0.0096), TRBV4‐2 (*p* = 0.03), TRBV4‐3(*p* = 0.048), TRBV5‐4(*p* = 0.011), TRBV6‐8(*p* = 0.038), TRBV7‐8 (*p* = 0.042), and TRBV7‐9(*p* = 0.023) usage showed significant difference. TRBV11‐2 (*p* = 0.016), TRBV12‐1 (*p* = 0.0068), TRBV16 (*p* = 0.0032), TRBV3‐2 (*p* = 0.0096), TRBV4‐2 (*p* = 0.03), TRBV4‐3 (*p* = 0.048), TRBV5‐4 (*p* = 0.011), TRBV6‐8 (*p* = 0.038), TRBV7‐8 (*p* = 0.042), and TRBV7‐9 (*p* = 0.023) usage showed significant difference. The higher usage genes provide the potential to target in specific immune‐related targeted medical approach.

In conclusion, we elucidated the different immunology repertoires in colorectal cancer patients and healthy controls. We further studied the effect of two anesthesia methods TIVA and CGEA on patients’ immune repertoires. We also studied TRBV and TRBJ genes which provided several potential targets for immune system‐targeted medicine for colorectal cancer. The immune repertoire will be a powerful tool for predicting the colorectal cancer surgery prognosis and identifying the targeted medicine.

## ETHICAL APPROVAL

This study was approved by the Institutional Review Board of the Department of Anaesthesiology, SHENZHEN PEOPLE'S HOSPITAL, 2nd Clinical Medical College of Jinan University, Shenzhen, Guangdong, China, in compliance with the Helsinki Declaration.

## CONFLICT OF INTERESTS

The authors have no conflict of interests to declare.

## AUTHORS' CONTRIBUTIONS

XL, YC, and YZ performed the experiments and wrote the manuscript. YL and ZZ made substantial contributions to conception, design, and intellectual content of the studies. ZL, QZ, WW, ZZ, and SL made key contributions to analysis and interpretation of data. All authors read and approved the final manuscript.

## Data Availability

The analyzed datasets generated during the study are available from the corresponding author on reasonable request.
